# Risk factors for nonidiopathic and idiopathic facial nerve palsies: findings of a retrospective study

**DOI:** 10.1186/s12883-024-03771-4

**Published:** 2024-07-26

**Authors:** Milena Kirchgässner, Samuel Böhm-Gonzalez, Johannes von Fraunberg, Benedict Kleiser, Stefanie Liebe, Christoph Kessler, Mihaly Sulyok, Alexander Grimm, Justus Marquetand

**Affiliations:** 1 Department of Epileptology, Hertie-Institute for Clinical Brain Research, University of Tübingen, Otfried-Müller-Str.47, 72076 Tübingen, Germany; 2 Department of Otolaryngology, Head and Neck Surgery, Tübingen Hearing Research Centre, Molecular Physiology of Hearing, University of Tübingen, Tübingen, Germany; 3 Department Neurodegenerative Diseases, Hertie-Institute for Clinical Brain Research, University of Tübingen, Tübingen, Germany; 4 Department of Pathology, University of Tübingen, Tübingen, Germany; 5 Department of Neural Dynamics and Magnetoencephalography, Hertie-Institute for Clinical Brain Research, University of Tübingen, Otfried-Müller-Str.47, 72076 Tübingen, Germany; 6 MEG-Center, University of Tübingen, Otfried-Müller-Str.47, 72076 Tübingen, Germany; 7 Institute for Modelling and Simulation of Biomechanical Systems, Stuttgart, Germany

**Keywords:** CSF, MRI, CT, Facial, Bell’s palsy

## Abstract

**Background:**

Idiopathic (IF) and nonidiopathic facial (NIF) nerve palsies are the most common forms of peripheral facial nerve palsies. Various risk factors for IF palsies, such as weather, have been explored, but such associations are sparse for NIF palsies, and it remains unclear whether certain diagnostic procedures, such as contrast agent-enhanced cerebral magnetic resonance imaging (cMRI), are helpful in the differential diagnosis of NIF vs. IF.

**Methods:**

In this retrospective, monocentric study over a five-year period, the medical reports of 343 patients with peripheral facial nerve palsy were analysed based on aetiology, sociodemographic factors, cardiovascular risk factors, consultation time, diagnostic procedures such as cMRI, and laboratory results. We also investigated whether weather conditions and German Google Trends data were associated with the occurrence of NIF. To assess the importance of doctors’ clinical opinions, the documented anamneses and clinical examination reports were presented and rated in a blinded fashion by five neurology residents to assess the likelihood of NIF.

**Results:**

A total of 254 patients (74%) had IF, and 89 patients (26%) had NIF. The most common aetiology among the NIF patients was the varicella zoster virus (VZV, 45%). Among the factors analysed, efflorescence (odds ratio (OR) 17.3) and rater agreement (OR 5.3) had the highest associations with NIF. The day of consultation (Friday, OR 3.6) and the cMRI findings of contrast enhancement of the facial nerve (OR 2.3) were also risk factors associated with NIF. In contrast, the local weather, Google Trends data, and cardiovascular risk factors were not associated with NIF.

**Conclusion:**

The findings of this retrospective study highlight the importance of patient history and careful inspections to identify skin lesions for the differential diagnosis of acute facial nerve palsy. Special caution is advised for hospital physicians during the tick season, as a surge in NIF cases can lead to a concomitant increase in IF cases, making it challenging to choose adequate diagnostic methods.

**Supplementary Information:**

The online version contains supplementary material available at 10.1186/s12883-024-03771-4.

## Background

Peripheral facial nerve palsy, which comprises both idiopathic and nonidiopathic forms, is a common neurological disorder. Idiopathic cases account for 60–75% of occurrences and lack a clearly defined cause, with current hypotheses suggesting a potential association with the activation of the herpes simplex virus in the facial nerve [1, 2]. Conversely, nonidiopathic facial nerve palsy (NIF) arises from a diverse range of factors, including infectious agents, neoplastic conditions, autoimmune disorders, and trauma. Despite advancements in understanding NIF, the pathogenesis of idiopathic facial nerve palsy (IF) remains elusive.

The predisposing risk factors identified for IF span a spectrum from pregnancy and autoimmune diseases to AIDS and cardiovascular comorbidities [3]. Notably, recent research has extended the exploration of risk factors to meteorological elements, such as temperature, wind strength, and chill [4]. Correlations between extreme temperatures and increased incidences of idiopathic peripheral facial nerve palsies, especially during cold seasons, have been discussed [5]. Wind temperature and strength have also been reported to be associated with a heightened probability of IF occurrence, with higher wind strengths and colder winds linked to increased instances of IF [6].

Idiopathic facial nerve palsy, commonly referred to as Bell’s palsy, represents the majority of acquired peripheral facial nerve palsies, accounting for 60–70% of cases [1, 3]. It is characterized by acute, usually unilateral, incomplete or complete paresis of the VII cranial nerve, with no externally apparent cause. The annual incidence of IF ranges from 7 to 40 cases per 100,000 inhabitants, affecting both genders equally [2]. In contrast, limited research has been conducted on the epidemiological and sociodemographic risk factors for nonidiopathic facial nerve palsy.

The literature notably lacks studies on the risk factors for various forms of nonidiopathic facial nerve palsies and predictor variables for the probability of NIF occurrence. Closing this gap is essential for a comprehensive understanding of the aetiologies of idiopathic and nonidiopathic facial nerve palsies as well as potential preventive measures.

## Methods

### Study design, patients, and procedures

**Fig. 1:**
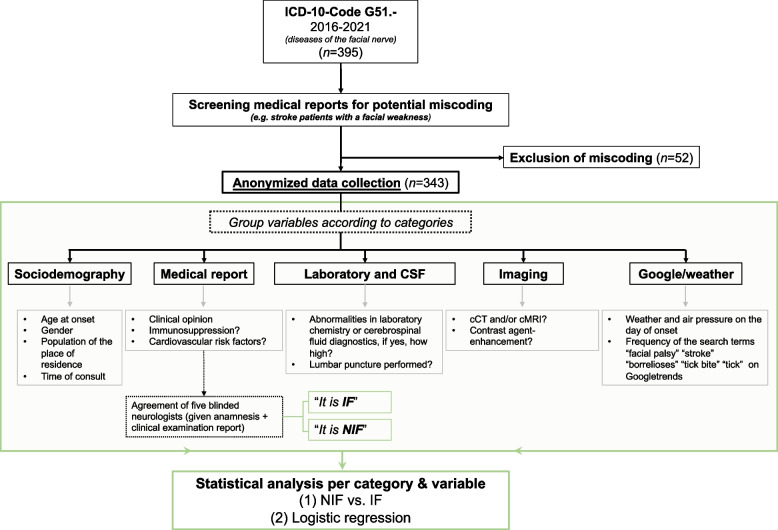
Flowchart of the data collection process. NIF: nonidiopathic facial palsy; IF: idiopathic facial palsy

The local electronic system at the University Hospital Tübingen was searched for the ICD-10 diagnosis G51 in the period from 16 March 2016 to 16 March 2021. Based on the available data (e.g. medical reports), an anonymized database of various variables was established (see Fig. 1 Fand Table 1).

**Table 1:**
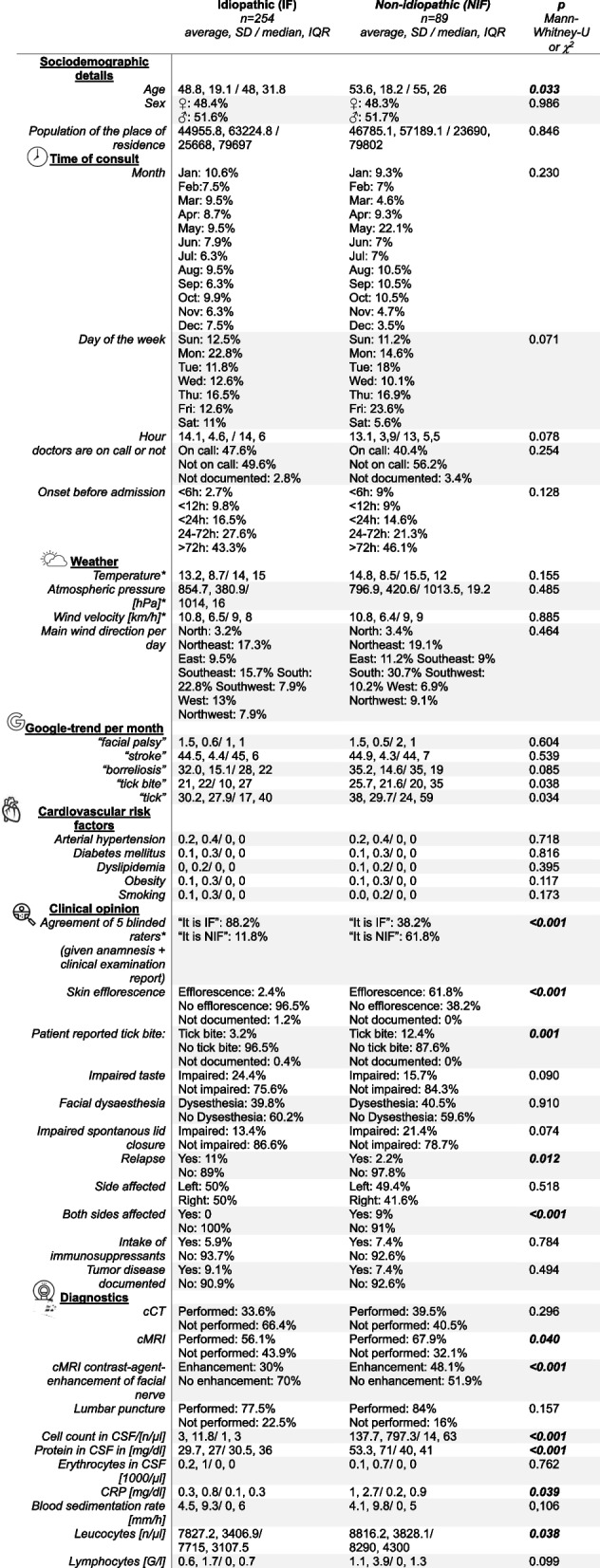
Description of average, standard deviation (SD) / median, inter quartile range (IQR) as well as statistical comparison of all variables assessed

Patients who were coded according to ICD-10 diagnosis G51 but did not have idiopathic or nonidiopathic facial palsies upon reviewing the medical documentation were excluded from further data collection (e.g. incorrectly coded facial nerve palsy in stroke patients). The sociodemographic parameters of the sample, including age at presentation and gender, were then analysed. Cardiovascular risk factors, such as arterial hypertension, nicotine abuse, diabetes mellitus, dyslipidaemia, and obesity, were also recorded. We also noted whether the peripheral facial nerve palsy was diagnosed as idiopathic or nonidiopathic in the discharge report, the cause of NIF, the affected side of the face (left, right, or both), and whether there was a recurrence. There was no follow up of the patients or observation of the progress. In addition, we documented whether the patients were under immunosuppression or had a known history of neoplasia. The year, month, day of the week, and time of presentation as well as the duration from symptom onset to admission at the University Hospital Tübingen were recorded. In addition, weather data such as temperature, air pressure, wind speed, and wind direction at the time of onset were through the website timeanddate (https://www.timeanddate.de/) collected. The patients' place of residence was specified as the weather location. Furthermore, we documented whether the patients were admitted during working hours (8:00 to 16:30) or outside of regular working hours (i.e., whether doctors were on call or not).

Finally, the German Google Trends data for the keywords “facial paralysis”, “stroke”, “ticks”, “tick bite”, and “Lyme disease” (in German language) in the 2016–2021 period were collected. In our study, we quantified the level of internet searching activity by utilizing data sourced from Google Trends. This online platform provides insights into the volume of Google internet searches for specific terms. The values obtained are indexed based on a scale where the period of maximum searching activity is assigned a value of 100. These indexed values were analyzed using a previously established methodology as described by Mavragani A and Ochoa G in their seminal paper on Google Trends' applications in infodemiology and infoveillance [7]. We extracted weekly data spanning from 2016 to 2021 for terms related to health conditions including “facial paralysis”, “stroke”, “ticks”, “tick bite”, and “Lyme Disease”, specifically using German keywords to reflect the search behaviors in Germany (general German population on Tuesday, not a patient visiting the hospital on Tuesday).

Regarding medical history, five raters (who were all medical staff at the hospital) conducted blind assessments of whether the reported symptoms were characteristic of idiopathic facial paresis. The list included the occurrence of a tick bite, taste disturbances, sensory disturbances, impaired eyelid closure, skin findings retro auricular, new deficits, and other abnormalities. There was no detailed descriptions of the palpation findings of the salivary gland in the doctor's letters. With respect to diagnostics, we noted whether the patients had undergone cranial computed tomography (cCT), cranial magnetic resonance imaging (cMRI), electrophysiological testing and/or lumbar puncture. The blood count data included the leukocytes, lymphocytes, C-reactive protein (CRP), and erythrocyte sedimentation rate (ESR), the CSF diagnostics included the cell count, proteins, and erythrocytes. If these were not present, any alternatives were documented. Since antibodies or PCR for a potential VZV infection were infrequently performed and also the location of skin lesions was not documented, we relied on the official diagnosis in the medical report, which was once again evaluated by an experienced neurologist (JM). Unless the clinical symptoms were explicitly described as “typical” in the doctors’ letters, the symptoms described in the literature [8] and guidelines “S2k-Leitlinie Therapie der Idiopathischen Fazialisparese (Bell’s palsy)” [2] and "Clinical Practice Guideline: Bell's Palsy" [9] were used as the criteria for the typical clinical manifestation of idiopathic facial nerve palsy (see also Supplement).

The collected data were tabulated and anonymized in a Microsoft Excel database. These were then imported into the statistical programs JASP 0.17.1 (JASP Team, 2022) and JMP 16.0 (SAS Institute Inc., Cary, NC). JASP 0.17.1 was used for performing logistic regressions and testing for differences between NIF and IF, while JMP 16.0 was used for visualization purposes.

The study design and procedures were approved by the local ethics committee of the University of Tuebingen, and a waiver of informed consent was obtained from the committee. The study was conducted in accordance with the World Medical Association’s Declaration of Helsinki.

### Data analysis and statistical procedures

Statistical analyses were performed using JMP 16.0 and JASP 0.17.1, and figures were illustrated using Microsoft PowerPoint. For both groups (NIF and IF), the distribution of data was first described using the average and standard deviation (SD) values as well as the median and interquartile range (IQR) values for all variables. Depending on the type of scale and the distribution (normal or non-normally distributed) of data in distinct categories (e.g. weather, CSF findings, and clinical opinions), the data were compared using the nonparametric Mann–Whitney U test or chi-square test. The Bonferroni correction was used to adjust the resulting *p*-values for multiple testing; accordingly, each *p*-value was multiplied by the number of tests per category (e.g. the *p*-value for day of the week was multiplied by seven, as there were seven tests).

Following the comparison of NIF and IF, a logistic regression analysis was employed to investigate the relationship between the predictor and binary outcome variables. The logistic regression model was chosen for its suitability in modelling the probability of an event occurring, given a set of variables per category (e.g. weather). The analysis was aimed at discerning the significance and strength of the associations between the selected predictors and thus obtaining valuable insights into the factors influencing the outcome variable.

## Results

**Fig. 2:**
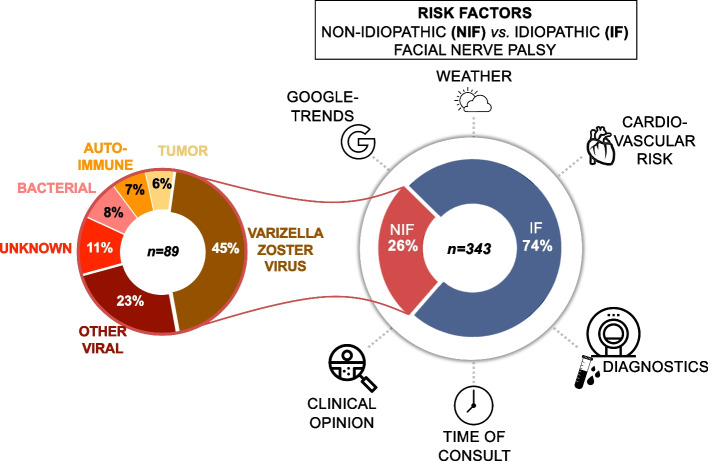
Graphical illustration using donut plots to visualize the study cohort, the variables, and the documented diagnoses of NIF in detail. NIF: nonidiopathic facial palsy; IF: idiopathic facial palsy

Of the 343 patients experiencing acute facial palsy, 89 (26%) exhibited NIF, while 254 (74%) presented with IF. Of all NIF cases, 9% (*n* = 8) presented with bilateral facial palsy. Viral infections, primarily those caused by the varicella zoster virus (VZV), were identified as the predominant causes of NIF (see Fig. 2). 18 (20%) borrelia cases were included in the infectious causes of NIF. It should be noted that tumours of the skull base had already been identified in 6% of the tumour diagnoses, which included T-cell lymphoma (*n* = 1), neurinoma of the facial knee (*n* = 1), Epidermoid cyst (*n* = 1) and not further specified tumours (*n* = 2). There was no case in which a tumour was first diagnosed because of acute NIF. Acute inflammatory demyelinating polyradiculoneuropathy (AIDP) (*n* = 1), chronic inflammatory demyelinating polyradiculoneuropathy (CIDP) (*n* = 4), Melkersson-Rosenthal syndrome (*n* = 1) were recorded under autoimmune diseases for NIF. Additional etiologies could not be found, and given that there was no scheduled follow-up visit for patients, we cannot provide any information about the course of NIF or IF in our cohort.

**Fig. 3:**
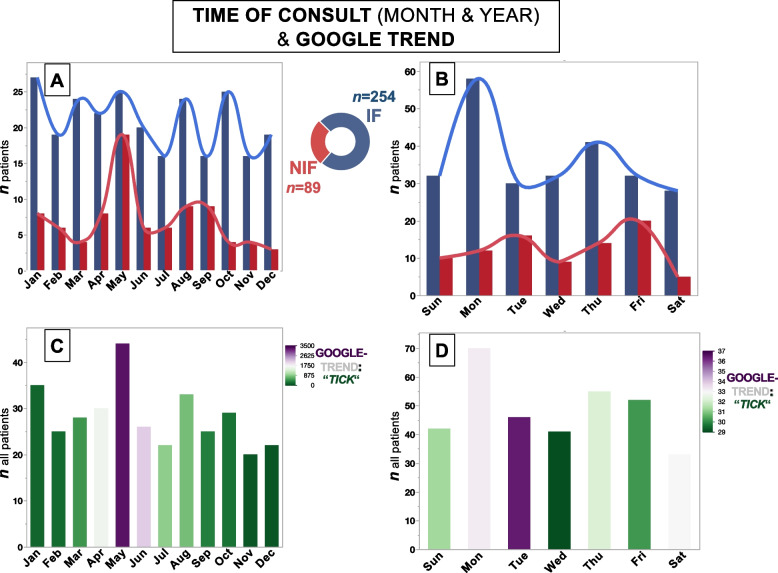
Bar charts of the consultation time and Google Trend results spanning the entire five-year dataset. NIF: nonidiopathic facial palsy; IF: idiopathic facial palsy. **A** The highest prevalence of NIF is in the month of May, whereas IF is randomly distributed across all months. **B** Prevalence of IF is highest on Monday, and that of NIF is on Friday. **C** The word “tick” (here German “Zecke”) had a similar high peak of searches (colour-scaled with purple; 3500 searches per month). **D** Patients who presented on a Tuesday (here, above patients) were also found to present in months (e.g. May) where “tick” was searched 37 times on average

Subsequently, we were interested in whether a Google Trends analysis of the aforementioned keywords would reveal a data distribution similar to the trend in the time of consult (month and year). We created a graphical representation of both trends across the entire five-year dataset, as depicted in Fig. 3. The highest prevalence of NIF is in the month of May and on Friday, whereas IF is randomly distributed across all months and most often on Monday; when performing a sub-analysis of VZV- and borreliosis cases in relation to the month of the year, we could observe a noticeable cluster of VZV in May and borreliosis in August (see Supplementary Fig. 1). The word “tick” (here German “Zecke”) had the highest peak of in May. Patients who presented on a Tuesday were also found to present in months (e.g. May) where “tick” was searched 37 times on average.

Detailed demographic results and comparisons of IF and NIF can be found in Table 1. Detailed results of the logistic regression of risk factors for NIF can be found in Supplementary Table 1. Among the various factors investigated, skin efflorescence (OR 17.3) and rater agreement (OR 5.3) showed the strongest associations with NIF. In addition, the day of consultation (Friday, OR 3.6) and contrast-enhanced cMRI findings of the facial nerve (OR 2.3) were identified as risk factors for NIF. Factors such as local weather, Google Trends data, electrophysiological testing, and cardiovascular risk were not associated with NIF; results of electrophysiological tests were not further evaluated.

## Discussion

In this retrospective, monocentric study of 343 patients with acute facial palsy, we investigated the characteristics and risk factors associated with NIF in contrast to those associated with IF. In line with previous epidemiological studies of NIF, viral infections were found to be the most prevalent underlying aetiology (68%; VZV dominant in 45% of all cases), and skin efflorescence was associated with a high risk of NIF (17-fold compared to IF). The novelty of our study lies not only in providing comprehensive reference data for variables involved in daily clinical routines but also in investigating previously unexplored variables, such as contrast enhancement of the facial nerve (OR 2.3), Google Trend data (not associated), and consultation time (Friday OR 3.6). Considering these insights, our study complements the characterization of NIF and clarifies the risk factors relevant to clinical practice, such as the intake of immunosuppressants, cardiovascular risk, and many others that do not increase the risk of NIF.

According to the findings of the clinical examination, almost two-thirds of the patients with NIF had efflorescence, whereas this was the case in only 2.4% of the IF patients. A limitation of the retrospective study design was that we were not able to characterize the efflorescence in IF patients any further. The risk of NIF in the presence of efflorescence was 17 times higher than that of IF, demonstrating the importance of thorough clinical examinations, including auricular examinations and microscopic examinations of the ear canal. From clinical experience, we determined that the differentiation between NIF and IF was not only based on the presence of efflorescence but also because of general medical opinions. We tried to reproduce the latter by conducting a blinded evaluation of the medical history documented in each patient’s medical report and the findings of the physical examination. Although one might assume that this approach would lead to a high true-positive rate for NIF based on the retrospective analysis, it turned out that 4 in 10 patients would have been misdiagnosed by the blinded raters (38.5% of the NIF cases diagnosed as IF). This underlines the results of previous assessments [1, 3] which showed that differential diagnosis is of high importance.

The difficulty of performing differential diagnoses in everyday clinical practice may have led to an excessive number of unnecessary diagnostics in our patient population, as there could be difficulty in differentiating between central and peripheral facial palsy in elderly patients with deep forehead wrinkles that make paralysis less noticeable, or a history of stroke (e.g., stroke directly affecting the facial nerve nucleus, also see exclusion criteria in the methods section). This may also explain the number of imaging diagnostics performed, even though these are known to be unnecessary in cases of acute facial palsy. A limitation in interpreting the reasons for the high number of imaging diagnostics is that the decision-making process for imaging was not documented in the medical reports and thus could not be retrieved. Notably, cMRI examinations were performed significantly more frequently in the NIF group than in the IF group, at 67.9% and 56.1% of cases, respectively. cCT examinations were performed less frequently overall but still in more than 30% of cases in both groups. The high number of contrast agents in the cMRIs provided us with information about the use of contrast enhancement to diagnose NIF and IF, which has not been investigated previously. Interestingly, contrast enhancement was more frequent for NIF than IF since inflammation was not locally limited in NIF cases, and this was pathophysiologically conclusive. However, the diagnostic utility of this approach is unclear at present, as enhancement has been reported in the literature in up to 91% of IF cases and even in 21% of a normal population without facial nerve palsy [10]. Nevertheless, there are clear justifications for imaging techniques in individual cases, especially to exclude acute central facial palsy or neoplastic genesis.

Although the heterogeneity of NIF cases poses a challenge to differential diagnosis, this makes the metadata all the more interesting. Possible influencing variables, such as climatic conditions, have already been discussed for IF but have not yet been investigated for NIF [11]. The distribution of IF (74%) and NIF (26%) cases in this study corresponded to the distribution in the population [11], with infectious diseases being the most common underlying aetiology of NIF, accounting for 76% of cases. According to the literature, the most common viral agent for facial nerve palsies is the varicella zoster virus [12]. In the case of an infection with varicella zoster virus, early initiation of adequate therapy is especially important for improving disease prognosis [13, 14].

We observed a trend in the month of the first presentation: almost one-third of the patients with NIF presented at the hospital in April and May, which is mainly due to an increase in VZV cases (Supplementary Fig. 1). Next to many possible explanations for this finding, we mainly see two possible reasons: (1) In Southern Germany, Borrelia burgdorferi is commonly transmitted by ticks and can lead to NIF, and the population and local practitioners are alerted to consult specialists during the tick season so that a higher rate of patients with VZV infections might have been sent to our hospital due to a fear of being infected with borreliosis (“attentional bias”, which might be reflected by the google search term “tick”, Fig. 3c). Considering the incubation time of Borrelia burgdorferi of weeks to a few months towards Neuroborreliosis, we could observe clustering of cases two up to three months after tick season, which seems to be a plausible finding. (2) Next to that, although pathophysiologically not fully understood yet, VZV infections seem to occur more frequently during summer [15–18]. We could also observe this circumstance for our cohort, which explains the increase in NIF cases during April and May. There were no significant differences in the month of first presentation between the IF and NIF groups in the cohort studied, which was surprising; an increase in IF cases in the period from March to May is typically not expected, but it was visible in our data. This three-month window accounted for 27.7% of all IF cases (Table 1), the highest for any three-month window within a year. In line with seasonal trends for NIF cases (see above), we offer two possible complementary explanations for this: As public awareness of ticks increases from March to May in Germany (see Fig. 3c), patients with facial paralysis are more likely to be referred to a university hospital to rule out the possibility of NIF, even if IF is more plausible. This may lead to an increase in the number of reported IF cases when NIF is indeed not diagnosed. In addition, the low rater agreement of 61.8% on NIF diagnoses (as opposed to 88.2% on IF diagnoses, Table 1) indicates that it is likely that misdiagnoses will occur, leading to additional IF cases during this period. Furthermore, and as discussed above it should be mentioned that VZV also shows a seasonal trend, which could indicate cases of IF sine herpete and thus explain the distribution of our data. NIF also occurred significantly more frequently on Fridays than IF did. Although this is probably of little clinical relevance and shouldn’t change the awareness of medical doctors for NIF on other days of the week, special attention should be paid towards of NIF on Fridays.

The epidemiological data in the present study population were correlated with the demand for the terms "facial palsy," "stroke," "borreliosis," "tick bite," and "tick" on Google Trends, but there was no significant association between the search trends and the occurrence of NIF or IF. In addition, the data showed no clear relationship between NIF or IF occurrence and the following factors: temperature, barometric pressure, and wind speed and direction.

Patient-specific risk factors such as diabetes mellitus and pregnancy have previously been reported for idiopathic facial nerve palsy [19]. Patient-specific risk factors for NIF were not found in the literature. In the present study population, arterial hypertension, diabetes mellitus, dyslipidaemia, obesity, and chronic nicotine use were not associated with clustered occurrence for NIF.

Detailed medical histories and thorough examination findings are of great importance in obtaining decisive findings in cases of IF or NIF (e.g., vesicles on erythematous ground, a tick bite in the recent past, a tumour of the parotid salivary gland). This was clearly demonstrated by the data collected in the present study.

## Conclusion

While IF is a common cranial nerve disease, NIF remains a frequent differential diagnosis that can be easily missed, and overdiagnostic measures in terms of imaging diagnostics do not seem to solve this challenge. The findings of this comprehensive retrospective study provide references for daily clinical practice.

### Supplementary Information

Additional file 1: Supplementary Fig. 1: Number of cases with VZV, borreliosis, and other NIF etiologies in relation to the month of the year. There is a noticeable cluster of VZV in May and Lyme disease in August. We refer to the discussion for possible explanations.

Additional file 2.

## Data Availability

No datasets were generated or analysed during the current study.

## Abbreviations

ESR: Erythrocyte sedimentation rate
CRP: C-reactive protein
CSF: Cerebrospinal fluid
CT: Computed tomography
IF: Idiopathic facial palsy (Bell’s palsy)
MRI: Magnetic resonance imaging
NIF: Nonidiopathic facial palsy

## References

[1] Heckmann JG, Urban PP, Pitz S, Guntinas-Lichius O, Gágyor I. The diagnosis and treatment of idiopathic facial paresis (Bell’s Palsy). Dtsch Arzteblatt Int. 2019;116:692–702. 10.3238/arztebl.2019.0692 PMC6865187 31709978

[2] Heckmann JG. Therapie der idiopathischen Fazialisparese. DGNeurologie. 2023;6(3):191–5. 10.1007/s42451-023-00549-9. 10.1007/s42451-023-00549-9

[3] Kim SJ, Lee HY. Acute peripheral facial palsy: recent guidelines and a systematic review of the literature. J Korean Med Sci. 2020;35:e245. 32743989 10.3346/jkms.2020.35.e245 PMC7402921

[4] Campbell KE, Brundage JF. Effects of Climate, Latitude, and Season on the Incidence of Bell’s Palsy in the US Armed Forces, October 1997 to September 1999. Am J Epidemiol. 2002;156:32–9. 12076886 10.1093/aje/kwf009

[5] De Diego JI, Prim MP, Madero R, Gavilán J. Seasonal patterns of idiopathic facial paralysis: a 16-year study. Otolaryngol Head Neck Surg Off J Am Acad Otolaryngol-Head Neck Surg. 1999;120:269–71. 10.1016/S0194-5998(99)70418-3 9949364

[6] Zhang W, Xu L, Luo T, Wu F, Zhao B, Li X. The etiology of Bell’s palsy: a review. J Neurol. 2020;267:1896–905. 30923934 10.1007/s00415-019-09282-4 PMC7320932

[7] Mavragani A, Ochoa G. Google trends in infodemiology and Infoveillance: methodology framework. JMIR Public Health Surveill. 2019;5:e13439. 31144671 10.2196/13439 PMC6660120

[8] Tiemstra JD, Khatkhate N. Bell’s palsy: diagnosis and management. Am Fam Physician. 2007;76:997–1002. 17956069

[9] Baugh RF, Basura GJ, Ishii LE, Schwartz SR, Drumheller CM, Burkholder R, et al. Clinical practice guideline: Bell’s palsy. Otolaryngol-Head Neck Surg Off J Am Acad Otolaryngol-Head Neck Surg. 2013;149(3 Suppl):S1-27. 10.1177/0194599813505967 24189771

[10] Kinoshita T, Ishii K, Okitsu T, Okudera T, Ogawa T. Facial nerve palsy: evaluation by contrast-enhanced MR imaging. Clin Radiol. 2001;56:926–32. 11603897 10.1053/crad.2001.0730

[11] Gilden DH. Clinical practice. Bell’s Palsy N Engl J Med. 2004;351:1323–31. 15385659 10.1056/NEJMcp041120

[12] Guntinas-Lichius O, Volk GF, Olsen KD, Mäkitie AA, Silver CE, Zafereo ME, et al. Facial nerve electrodiagnostics for patients with facial palsy: a clinical practice guideline. Eur Arch Oto-Rhino-Laryngol Off J Eur Fed Oto-Rhino-Laryngol Soc EUFOS Affil Ger Soc Oto-Rhino-Laryngol - Head Neck Surg. 2020;277:1855–74. 10.1007/s00405-020-05949-1 PMC7286870 32270328

[13] Murakami S, Hato N, Horiuchi J, Honda N, Gyo K, Yanagihara N. Treatment of Ramsay Hunt syndrome with acyclovir-prednisone: significance of early diagnosis and treatment. Ann Neurol. 1997;41:353–7. 9066356 10.1002/ana.410410310

[14] Monsanto R da C, Bittencourt AG, Bobato Neto NJ, Beilke SCA, Lorenzetti FTM, Salomone R. Treatment and Prognosis of Facial Palsy on Ramsay Hunt Syndrome: Results Based on a Review of the Literature. Int Arch Otorhinolaryngol. 2016;20:394–400. 27746846 10.1055/s-0036-1584267 PMC5063726

[15] Vergara-Castañeda A, Escobar-Gutiérrez A, Ruiz-Tovar K, Sotelo J, Ordoñez G, Cruz-Rivera MY, et al. Epidemiology of varicella in Mexico. J Clin Virol Off Publ Pan Am Soc Clin Virol. 2012;55:51–7. 10.1016/j.jcv.2012.06.004 22750018

[16] Jung HS, Kang JK, Yoo SH. Epidemiological Study on the Incidence of Herpes Zoster in Nearby Cheonan. Korean J Pain. 2015;28:193–7. 26175879 10.3344/kjp.2015.28.3.193 PMC4500783

[17] Khaleel HA, Abdelhussein HM. Clinical epidemiology of chickenpox in Iraq from 2007–2011. Glob J Health Sci. 2012;5:180–6. 23283051 10.5539/gjhs.v5n1p180 PMC4776987

[18] Miller ER, Kelly HA. Varicella infection – Evidence for peak activity in summer months. J Infect. 2008;56:360–5. 18359087 10.1016/j.jinf.2008.01.050

[19] Warner MJ, Hutchison J, Varacallo M. Bell Palsy. In: StatPearls. Treasure Island: StatPearls Publishing; 2023.

