# Supplementary material for: Risk factors for nonidiopathic and idiopathic facial nerve palsies: findings of a retrospective study
Source: BMC Neurol. 2024 Jul 26;24:259. doi: 10.1186/s12883-024-03771-4 (PMC11282606; doi:10.1186/s12883-024-03771-4)
Supplement: Supplementary file 1 — Additional file 1: Supplementary Fig. 1: Number of cases with VZV, borreliosis, and other NIF etiologies in relation to the month of the year. There is a noticeable cluster of VZV in May and Lyme disease in August. We refer to the discussion for possible explanations. [file 12883_2024_3771_MOESM1_ESM.docx]

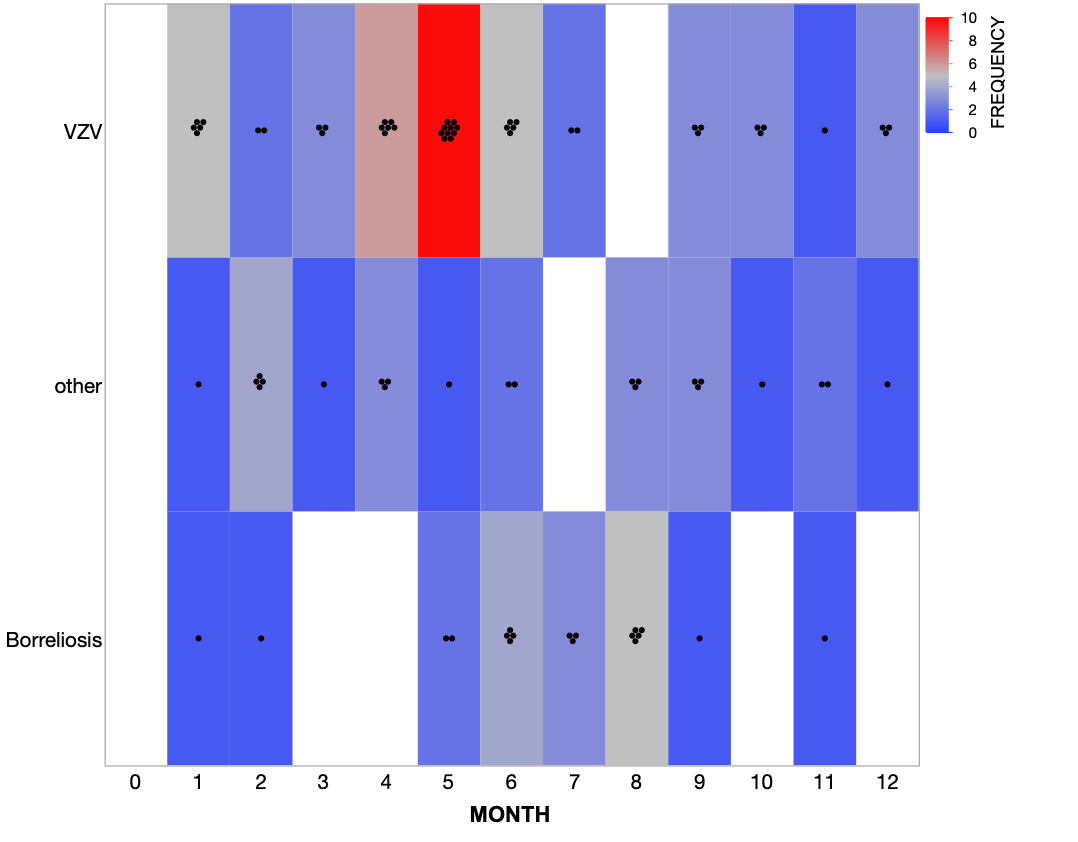


***Supplementary Figure 1:*** Number of cases with VZV, borreliosis, and other NIF etiologies in relation to the month of the year. There is a noticeable cluster of VZV in May and Lyme disease in August. We refer to the discussion for possible explanations.
