# Supplementary material for: Risk factors for nonidiopathic and idiopathic facial nerve palsies: findings of a retrospective study
Source: BMC Neurol. 2024 Jul 26;24:259. doi: 10.1186/s12883-024-03771-4 (PMC11282606; doi:10.1186/s12883-024-03771-4)

**Supplement**

|  | **Odds ratio** | **95% confidence interval** | **p**  (Wald-Test) |
| --- | --- | --- | --- |
| **Sociodemographic details** |  |  |  |
| Age | ***1*** | ***1–1*** | ***0.038*** |
| Sex | 1 | 0.6–1 | 0.614 |
| Population of the place of residence | 1 | 1–1 | 0.706 |
| (Intercept) | 0.1 | 0.1–0.3 | < 0.001 |
| 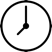**Consultation time** |  |  |  |
| Month  (Jan = intercept) | Feb: 0.8  Mar: 0.3  Apr: 1.4  May: 1.7  Jun: 0.9  Jul: 0.9  Aug: 0.9  Sep: 1.3  Oct: 0.5  Nov: 0.4  Dec: 0.5 | 0.2–2.8  0.1–2.8  0.5–4.6  0.6–5.1  0.2–3.2  0.2–3.3  0.3–2.7  0.4–4.5  0.1–2  0.1–2.2  0.1–2.3 | 0.230  0.360  0.537  0.480  0.641  0.998  0.346  0.582  0.746  0.988  0.816 |
| Day of the week  (Wed = intercept) | Sun: 1.3  Mon: 1  Tue: 2.2  Thu: 1.8  **Fri: 3.6**  Sat: 0.6 | 0.4–4.4  0.4–3.2  0.7–6.5  0.6–5.4  **1.2–10.5**  0.2–2.7 | 0.680  0.953  0.177  0.267  ***0.019***  0.556 |
| Hour | 0.9 | 0.1–1.9 | 0.057 |
| on call or not | On call: 1.3 | On call: 0.6–2.6 | 0.480 |
| (Intercept) | 0.5 | 0.1–2 | 0.310 |
| 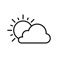**Weather** |  |  |  |
| Temperature* | 1 | 1–1 | 0.139 |
| Atmospheric pressure* | 1 | 1–1 | 0.078 |
| Wind velocity * | 1 | 1–1.1 | 0.209 |
| Main wind direction per day  (North = intercept) | Northeast: 1  East: 1.2  Southeast: 0.5 South: 1.9 Southwest: 1.2  West: 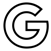1.2  Northwest: 1.5 | 0.2–5.6  0.2–7.3  0.1–3.4  0.4–10.2  0.2–7.6  0.1–3.7  0.3–8.9 | 0.971  0.842  0.507  0.478  0.829  0.565  0.682 |
| (Intercept) | 0.3 | 0.1–1.3 | 0.093 |
| **Google Trends data per month** |  |  |  |
| “facial palsy” | 1.2 | 0.1–0.74 | 0.547 |
| “stroke” | 1 | 1–1 | 0.444 |
| “borreliosis” | 1 | 1–1 | 0.107 |
| “tick bite” | 1 | 1–1 | 0.265 |
| “tick” | 1 | 1–1 | 0.240 |
| 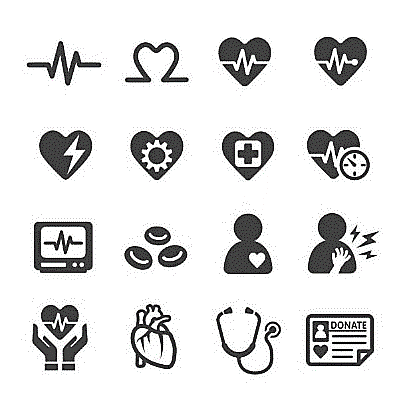(Intercept) | 0.1 | 0.1–0.7 | 0.032 |
| **Cardiovascular risk factors** |  |  |  |
| Arterial hypertension | 1.2 | 0.6–2.2 | 0.718 |
| Diabetes mellitus | 0.6 | 0.2–1.7 | 0.816 |
| Dyslipidaemia | 1.6 | 0.5–6 | 0.395 |
| Obesity | 1.5 | 0.6–3.5 | 0.117 |
| Smoking | 0.4 | 0.6–2.2 | 0.173 |
| (Intercept) | 0.3 | 0.2–0.4 | < 0.001 |
| 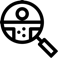**Clinical opinion** |  |  |  |
| Agreement of five blinded raters*  (given anamnesis + clinical examination report) | ***“It is NIF”: 5.2*** | ***2.6–10.3*** | ***< 0.001*** |
| Skin efflorescence | ***17.3*** | ***6.4–47.2*** | ***< 0.001*** |
| Patient reported tick bite: | 1.5 | 0.4–5.5 | 0.584 |
| Impaired taste | 0.9 | 0.4–2.1 | 0.842 |
| Facial dysaesthesia | 1.1 | 0.6–2.2 | 0.706 |
| Impaired spontaneous lid closure | 1.8 | 0.8–4 | 0.172 |
| Relapse | 0.9 | 0.7–3.3 | 0.106 |
| Intake of immunosuppressants | 0.9 | 0.1–3.3 | 0.784 |
| Tumour disease documented | 1.5 | 0.4–5.6 | 0.52 |
| (Intercept) | 0.1 | 0.1–0.2 | < 0.001 |
| 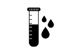**Diagnostics** |  |  |  |
| cMRI contrast agent-enhancement of facial nerve | ***2.3*** | ***1.4–3.9*** | ***0.002*** |
| CRP | ***1.6*** | ***1.1–2.3*** | ***0.011*** |
| Blood sedimentation rate | 1 | 1.1–2.3 | 0.084 |
| Leukocytes | 1 | 1–1 | 0.294 |
| Lymphocytes | 1.1 | 1–1.2 | 0.079 |
| (Intercept) | 0.1 | 0.1–0.3 | < 0.001 |


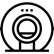

Supplement: Supplementary file 2 — Additional file 2. [file 12883_2024_3771_MOESM2_ESM.docx]
